# The Effectiveness, Costs and Coastal Protection Benefits of Natural and Nature-Based Defences

**DOI:** 10.1371/journal.pone.0154735

**Published:** 2016-05-02

**Authors:** Siddharth Narayan, Michael W. Beck, Borja G. Reguero, Iñigo J. Losada, Bregje van Wesenbeeck, Nigel Pontee, James N. Sanchirico, Jane Carter Ingram, Glenn-Marie Lange, Kelly A. Burks-Copes

**Affiliations:** 1 National Center for Ecological Analysis and Synthesis (NCEAS), University of California Santa Barbara, Santa Barbara, California, United States of America; 2 The Nature Conservancy / University of California Santa Cruz, Santa Cruz, California, United States of America; 3 Department of Ocean Sciences, UC Santa Cruz / The Nature Conservancy, Santa Cruz, California, United States of America; 4 Instituto de Hidraulica Ambiental, ETSI de Caminos, Canales y Puertos, Universidad de Cantabria, Santander, Spain; 5 Unit for Marine and Coastal Systems, Deltares / Delft University of Technology, Delft, The Netherlands; 6 Coastal Planning and Engineering, CH2M HILL / University of Southampton, Swindon, United Kingdom; 7 Department of Environmental Science and Policy, University of California Davis, Davis, California, United States of America; 8 Wildlife Conservation Society / Columbia University, New York, New York, United States of America; 9 Environment and Natural Resources Global Practice, The World Bank, Washington, DC, United States of America; 10 Environmental Laboratory, US Army Engineer Research and Development Center, Vicksburg, Mississippi, United States of America; University of Sydney, AUSTRALIA

## Abstract

There is great interest in the restoration and conservation of coastal habitats for protection from flooding and erosion. This is evidenced by the growing number of analyses and reviews of the effectiveness of habitats as natural defences and increasing funding world-wide for nature-based defences–*i*.*e*. restoration projects aimed at coastal protection; yet, there is no synthetic information on what kinds of projects are effective and cost effective for this purpose. This paper addresses two issues critical for designing restoration projects for coastal protection: (i) a synthesis of the costs and benefits of projects designed for coastal protection (nature-based defences) and (ii) analyses of the effectiveness of coastal habitats (natural defences) in reducing wave heights and the biophysical parameters that influence this effectiveness. We (i) analyse data from sixty-nine field measurements in coastal habitats globally and examine measures of effectiveness of mangroves, salt-marshes, coral reefs and seagrass/kelp beds for wave height reduction; (ii) synthesise the costs and coastal protection benefits of fifty-two nature-based defence projects and; (iii) estimate the benefits of each restoration project by combining information on restoration costs with data from nearby field measurements. The analyses of field measurements show that coastal habitats have significant potential for reducing wave heights that varies by habitat and site. In general, coral reefs and salt-marshes have the highest overall potential. Habitat effectiveness is influenced by: a) the ratios of wave height-to-water depth and habitat width-to-wavelength in coral reefs; and b) the ratio of vegetation height-to-water depth in salt-marshes. The comparison of costs of nature-based defence projects and engineering structures show that salt-marshes and mangroves can be two to five times cheaper than a submerged breakwater for wave heights up to half a metre and, within their limits, become more cost effective at greater depths. Nature-based defence projects also report benefits ranging from reductions in storm damage to reductions in coastal structure costs.

## Introduction

Tens of millions of people world-wide will be affected in the next few decades by coastal flooding due to sea-level rise and associated increases in wave action and surges [1,2]. In addition, coastal habitats are facing increasing risks world-wide as a result of human activity. These habitats provide a number of ecosystem services, or benefits, including coastal protection, fish production, recreation and other economic and cultural values [3]. In many instances the degradation of coastal habitats can result in a decrease in coastal protection and increase risk of coastal flooding [4,5]. Observations of extreme events like the Indian Ocean tsunami, Hurricanes Sandy and Katrina in the Atlantic and Typhoon Haiyan in the Pacific suggest that coastal habitats can help protect coastlines during such events [6,7,8,9]. There is hence great interest in identifying effective, and cost effective solutions that help conserve habitats and protect coastlines [10,11].

While there is important interest in the conservation of habitats for the natural defence they provide, there is a particularly strong interest in investments in restoring coastal habitats for nature-based defences. In this paper, natural defences refer to existing coastal habitats within which wave reduction has been measured. Nature-based defences refer to restoration projects that specifically include coastal protection as an objective (definitions adapted from the U.S. Army Corps of Engineers report on Natural and Nature-based Features [12]). A number of restoration projects world-wide are being implemented specifically for coastal protection [13]. These are increasingly driven by global conventions and their funding mechanisms, including the United Nations Framework Convention on Climate Change (UNFCCC) and the green Adaptation Fund (AF), as well as the United Nations International Strategy for Disaster Risk Reduction (UNISDR) and lending from the World Bank. They are also being driven by interest from national and multi-national agencies [10,12,14,15]. But critical questions remain about when these projects can be used effectively for coastal protection, for example about the costs of a habitat restoration project relative to other, more conventional alternatives.

The contribution of habitats to coastal protection is increasingly addressed in science, policy and practice [16,17,18]. There is also a growing interest in developing guidance about habitat restoration for nature-based defences but this has largely been identified based more on case studies than syntheses [12,18,19]. Insights on the success or failure of projects, and comparisons with hard structures that perform similarly, under the same conditions, are difficult to obtain [20]. While there are a number of studies analysing the physical aspects of coastal protection by coastal habitats, there is very little information to date that combines this knowledge with information on restoration projects, to assess the performance and viability of these projects. This can hinder decision-making with regard to future investments in restoration projects for coastal protection.

Widespread consideration and use of habitats for coastal protection still face significant challenges including: a) uncertainty around the effectiveness of habitats under different hydrodynamic and ecological conditions; b) a lack of synthetic information about the costs and effectiveness of projects that restore or manage habitats for coastal protection; and c) a paucity of studies that integrate and synthesise engineering and ecological knowledge to provide site-specific comparisons of the costs and effectiveness of nature-based defences versus hard structures. This paper integrates analyses of (a) natural defences with information from (b) nature-based defence projects, to address these gaps and improve understanding of how and where coastal habitats may be viable for coastal protection.

**Fig 1:**
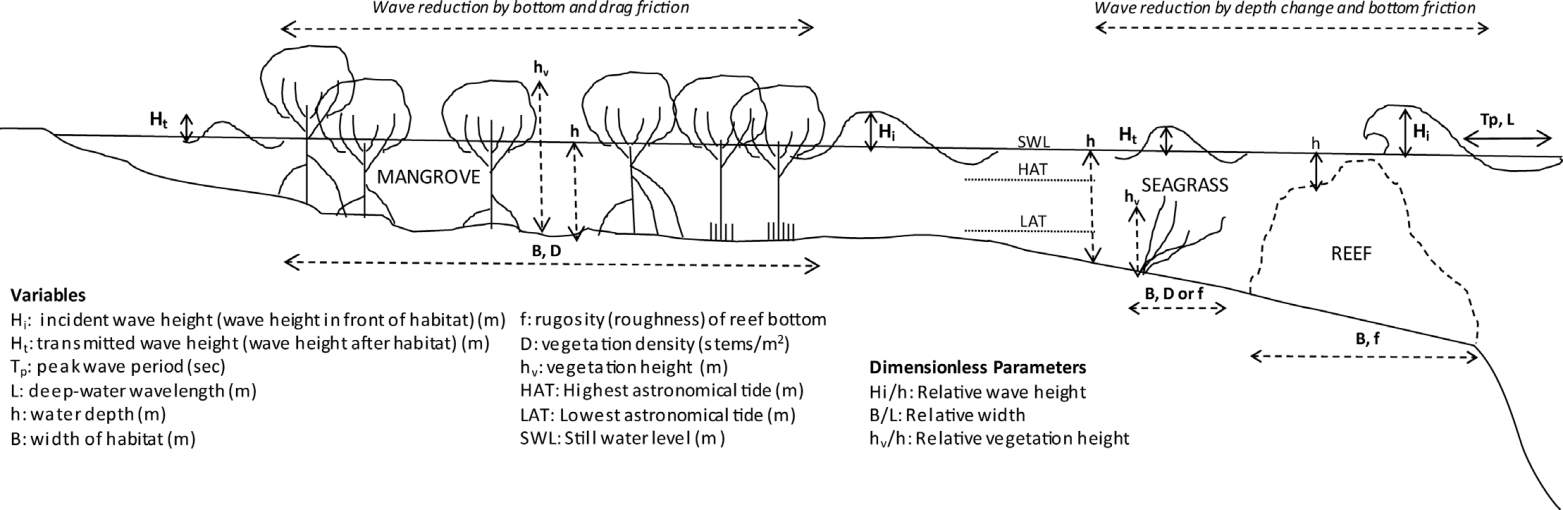
Schematic of wave height reduction across coastal habitats. Schematic showing general mechanics of wave height reduction through habitats, using the examples of coral reefs, seagrass beds and mangroves.

This is done by: a) analysing field measurements of wave reduction within coastal habitats and the parameters that may drive variability in this function; and b) mapping and combining information from these field measurements with information on nearby nature-based defence projects, to compare their costs with hard structures that will provide the same level of wave reduction. First we extend previous syntheses of wave reduction field measurements in coastal habitats [21,22,23] to include more measurements and improve understanding of the variability across habitats in reducing wave heights, focusing in particular on engineering parameters that will be critical in assessing and designing restoration projects. In their re-analyses of field data, Pinsky et al., [23] show high variability in wave reduction between habitats and investigate the influence of biophysical parameters on this variability–namely, the local flow conditions (Reynold’s number) and the resistance to flow provided by the habitat (the bulk drag coefficient). In our study, data from field measurements are used to directly investigate the influence of biophysical parameters on this variability (Fig 1). The field measurements are then mapped and, based on their location and habitat type, are combined with details of nearby nature-based defence projects. These nature-based defence projects are first analysed for their costs and benefits for coastal protection. Based on information from nearby field measurements, wave reduction extents are estimated for some of these projects and their costs compared to the costs of submerged breakwaters that will provide the same wave height reduction under the same conditions. These results provide insights on where and how coastal habitats and nature-based defence projects may be viable and cost effective, and also, on the key parameters that should be assessed when designing these projects.

## Results and Discussion

**Fig 2:**
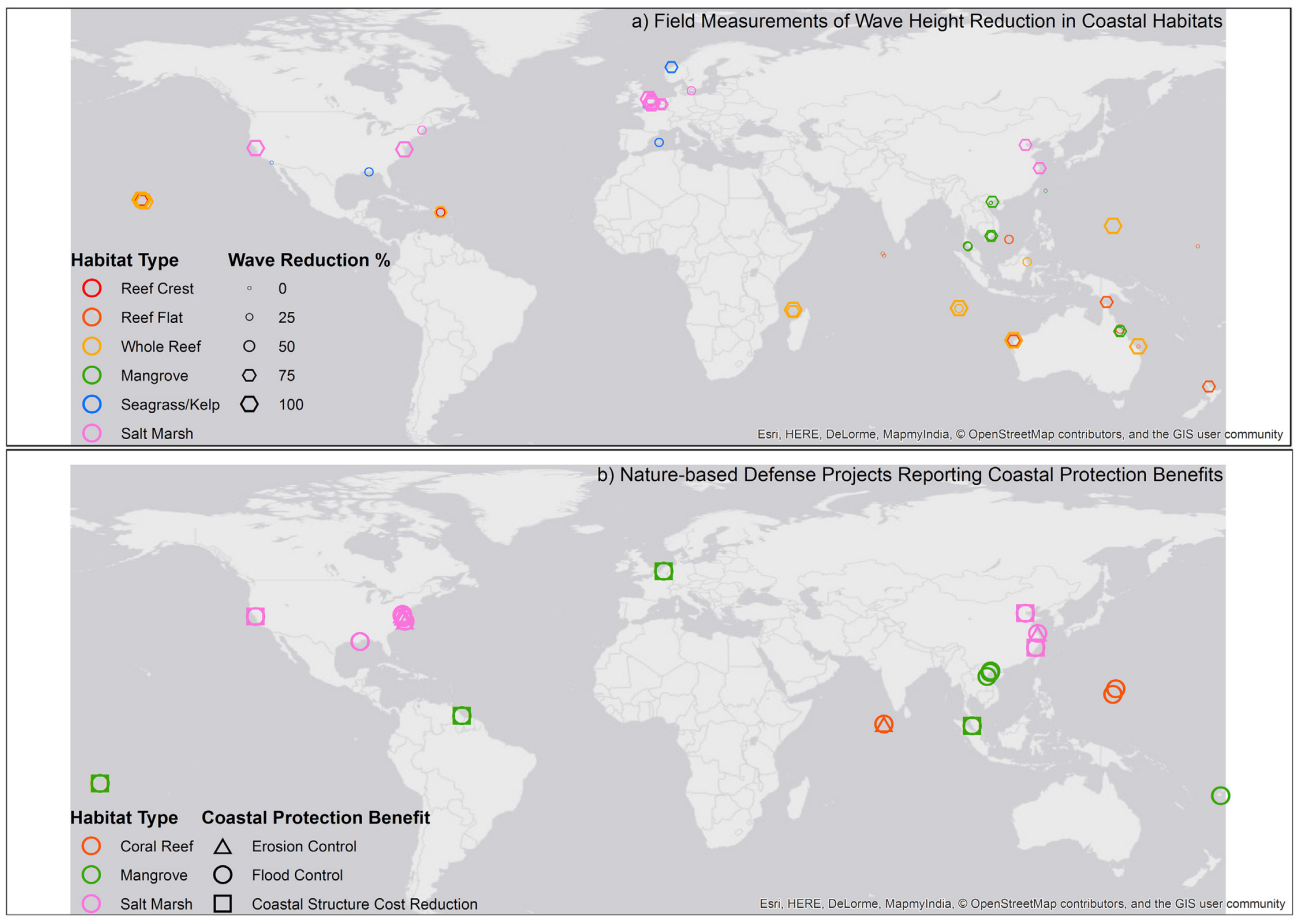
**Global map of a) wave height reduction in natural defences (n = 69) and b) Coastal protection benefits from restoration projects (n = 52).** Panel (a) maps wave height reduction measurements in coral reefs, salt marshes, mangroves, seagrass beds, kelp beds; Panel (b) maps restoration projects reporting coastal protection benefits reviewed for coral reefs, salt marshes and mangroves (the literature search did not find information on oyster reef projects that observe coastal protection benefits). Colours indicate habitat groups in both panels. Circle sizes in (a) indicate the % wave height reduction measured at each site; shapes in (b) indicate type of coastal protection benefit reported (erosion control, flood control, or protection to structures) (see Table 1). Basemap image is the intellectual property of Esri and is reprinted from Esri under a CC BY license with permission from Esri and its licensors, all rights reserved. Credits: Esri, HERE, DeLorme, NGA, USGS | Esri, HERE, DeLorme.

This paper analyses sixty-nine field measurements to show that habitats have a significant influence on wave reduction, demonstrates the influence of specific engineering parameters on wave reduction effectiveness, reviews the costs and benefits of fifty-two nature-based defence projects (Fig 2), and demonstrates the cost-effectiveness of some of these projects relative to structures that provide the same wave reduction.

### Natural defences and wave reduction

Meta-analyses of sixty-nine studies, among five habitats world-wide (coral reefs, mangroves, salt-marshes, seagrass/kelp beds), show that these habitats reduce wave heights significantly (see Methods) and this reduction varies with the habitat and the site. This is in line with findings from [21] and [23]. On average, coastal habitats reduce wave heights between 35% and 71%. Coral reefs reduce wave heights by 70% (*95% CI*: *54–81%*), salt-marshes by 72% (*95%CI*: *62–79%*), mangroves by 31% (*95% CI*: *25–37%*) and seagrass/kelp beds by 36% (*95% CI*: *25–45%*). Across all habitats, coral reefs emerge as having the greatest potential for coastal protection: they are highly effective at reducing wave heights and are also exposed to higher, more powerful waves. Salt-marshes are almost as effective in terms of wave reduction but occur in more sheltered environments. Mangroves and seagrass / kelp beds are about half as effective, with mangroves occurring in the most sheltered environments (see S1 Table). The high reduction by coral reefs agrees with the results of [21], and the ordering of the other habitats is generally in agreement with the review by [23] which considered similar parameters in their re-analyses of field evidence for these habitats. There is also a strong positive, linear correlation between the extent of reductions in wave height, and the wave height before the habitat, in the order coral reefs > salt-marshes ~ mangroves > seagrass / kelp beds (see S1 Fig).

The influence of design parameters commonly used in engineering such as habitat width, the ratio of habitat width to the wavelength, and the ratio of habitat height to the water level (see Introduction, Fig 1) were also examined. Wave reduction in each habitat is influenced by different parameters. In coral reefs, wave reduction is influenced by a) reef width (S2 Fig); b) reef depth relative to the wave height and; c) reef width relative to the average wavelength (S3 Fig). The most effective reefs are at least twice as wide as the wave-length, and located at depths that are at most, half the incoming wave height. There is limited data in salt-marshes that suggests that wave reduction is linearly correlated with the relative height of the marsh, i.e. the submergence of the vegetation relative to the water level (S4 Fig). Wave reduction in salt-marshes is highest when the canopy is close to the water surface. This suggests that designs of ‘greenbelts’ for coastal protection, rather than only relying on width-based criteria [24,25], should also account for the relationships between habitat and hydrodynamic variables at each site. is also emphasised by Koch et al., [26] who demonstrate spatial and temporal variations in wave reduction capacities across habitats. These analyses were performed only for coral reef and salt-marsh habitats. Mangroves and seagrass/kelp beds are not discussed due to the lack of comparable data on design parameters for these habitats.

### Nature-based defence projects: costs, benefits and cost effectiveness for coastal protection

Table 1 summarises the costs, coastal protection benefits, objectives and exposure of fifty-two nature-based defence projects in coral reef, oyster reef, mangrove, and salt-marsh habitats. A sizeable proportion of salt-marsh and mangrove projects state that such habitats provide improved protection from storm events (41% in salt-marshes and 50% in mangroves; see Table 1). Other coastal protection benefits include savings in damages during storm events, reductions in erosion and reductions in the costs of engineering for coastal protection, reflected, in a few cases, by positive benefit-cost ratios (e.g. also see [20]). Restoration objectives vary across habitat types, with most mangrove and marsh habitats reporting coastal protection as a primary objective. Among the coral reefs a majority of projects are targeted primarily at habitat restoration and only a small number for coastal protection, even though many of these reefs are situated in highly exposed regions. Unit restoration costs are lowest for marshes and mangroves, and coral and oyster reefs show higher, and more variable, costs (Table 1).

**Table 1:** Costs and Coastal Protection Benefits of Restoration Projects.

Habitat	Reported Restoration Project Costs^ as US $ Per m^2^: Median (Range)	Estimated Replacement Cost Ratios*: Average (*95% CI*)	% of Projects implemented for coastal protection	% of Projects in High Exposure Regions ^#^	% of Projects reporting coastal protection benefits^✞^
Coral Reefs (*n = 19*)	115.62 (2–7490)	NA	5	80	ER– 5; FL– 5
Oyster Reefs (*n = 4*)	135.63 (107–316)	NA	75	50	NA
Salt-Marshes (*n = 17*)	1.11 (0.01–33)	2 (0.95–3.01)	69	77	ER– 6; FL– 41; ST– 18; BC– 6
Mangroves (*n = 12*)	0.1 (0.05–6.43)	5 (3.1–6.9)	76	35	FL– 50; ST– 34; BC– 41

n = total no. of projects for each habitat type. CI = confidence interval.

^: Project costs not scaled; areas for which costs are reported vary across studies (see S3 Table).

*: Replacement cost ratio = submerged breakwater cost / nature-based defence cost (see Methods).

#: High exposure regions defined as regions with > 10 J/m^2^ average annual wave energy based on global deep-water wave climate dataset in [44].

✞: Coastal protection benefit types = ER–savings in erosion damage costs; FL–savings in damages costs from storms; ST–savings in costs of adjacent coastal structures; BC–project benefit / cost ratio > 1.

Note: some projects report multiple benefits (see S3 Table).

**Fig 3:**
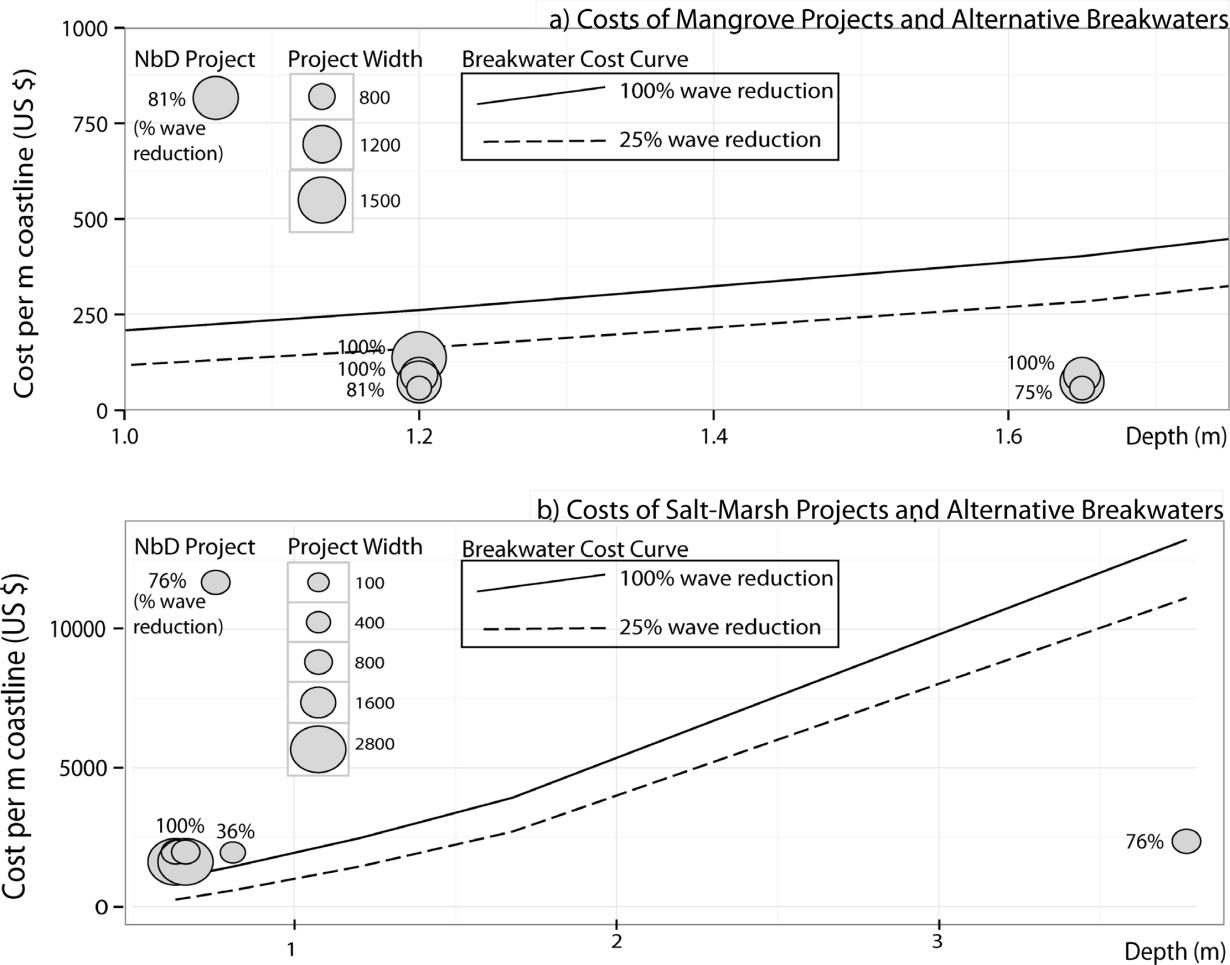
Costs versus water depth and wave height reduction extents of Nature-based Defence (NbD) projects and alternative breakwaters. Costs of NbDs and cost curves of alternative breakwater structures plotted versus water depth are plotted for a) mangroves (n = 7) and breakwaters in Vietnam and; b) salt-marshes (n = 6) and breakwaters in Europe/USA. Circles represent NbDs and lines represent submerged breakwaters cost-curves in both panels. NbDs that fall below breakwater cost curves are cost effective in comparison. Breakwater cost curves are for an incident wave height Hsi of 0.2 m. All costs are represented on a per-metre coastline length basis (see Methods). Fig only shows mangroves and marshes as these were the only habitat types and locations for which project information was found in close proximity to field measurements.

Analyses of the costs and wave reduction of thirteen nature-based defence projects (see Methods, S4 Table) in mangroves and salt-marshes show that these projects can be several times cheaper than alternative submerged breakwaters (Fig 3) for the same level of protection. Together with their ability to keep pace with sea-level rise [27] this suggests that nature-based defences can become increasingly viable on sheltered coastlines. Depending on the water depth, mangrove projects in Vietnam can be three to five times cheaper than a breakwater, and salt-marsh projects across Europe and the USA vary from being just as expensive, to around three times cheaper (Table 1, Fig 3). Fig 3 plots the total restoration costs of mangrove and marsh projects along with breakwater construction costs at these sites for a range of depths and wave height reduction values. The cross-shore width of habitat and the degree of wave height reduction are also indicated for each project. Water depth is a crucial factor, with both habitats showing an increase in cost effectiveness at higher depths, due to the relatively steep increase in breakwater construction costs. Habitat width is found not to be a sufficient indicator of cost effectiveness. Also, these nature-based defences are limited to wave heights less than half a metre and are not always cost effective.

Based on existing literature it was assumed that breakwater construction costs are uniform across the sites in Europe/USA and ten times lower for the sites in Vietnam [28]. Such regional differences are also reflected in the reported habitat restoration costs in these countries. While accurate estimates of construction costs require detailed information on structure profile, material and labour costs, etc., water depth is often a critical driver of construction costs [29] and therefore the main influence on cost effectiveness. Only total project costs and habitat extents were used, given the high variability in the relationship between restoration project costs and sizes (see Methods). The study does not explicitly account for increases in restoration costs due to adverse ecological or geomorphic site conditions which can significantly increase these values [30].

In the cost comparisons we look for structures that are equivalent to marshes and mangroves in function–i.e. wave reduction, as well as location–i.e. within the near-shore zone, and choose submerged breakwaters as the best alternative. Submerged breakwaters provide wave reduction to varying degrees, similar to coastal habitats and can be located within the near-shore zone. Though seawalls are a common substitute for mangrove and marsh habitats [20,31], these are often located at the shoreline and, in addition to blocking waves, also protect against flooding from high water levels. While there are some indications that mangroves and marshes can offer protection from high water levels (Table 1), we do not find enough evidence on this for a comparison of effectiveness, and as such, focus on their wave reduction function. While coral reefs are also very similar to breakwaters in structure and wave reduction function, we do not find enough information on reef restoration projects for a direct cost comparison. It is important to note that coastal habitats are usually one of several structural, nature-based and non-structural measures for coastal protection [32].

This study focuses on coastal protection by wave reduction, though habitats often provide other ecosystem services such as biodiversity, fish production, recreation and many other social, economic and cultural values [33]. The addition of these benefits, over and above their coastal protection value should make these natural approaches more appealing to coastal managers and decision-makers [34]. Also, the loss of existing coastal habitats and their replacement by man-made structures can result in loss of these ecosystem services [35]. In any case, policy decisions on where and how to conserve or restore habitats, rather than focusing on a single service, should consider multiple objectives for best allocation of available resources [36,37].

The data for the wave reduction analyses are all obtained from field observations of wave heights and hydrological variables. The datasets used in this study vary in terms of the type of data available for analysis, and these are described in S2 and S3 Tables. The wave reduction data are all field observations of wave heights through habitats (S2 Table). Almost all the studies provide information on habitat width, and most measurements in reefs also provide information on reef depth. Only a few studies–all in marshes, provide information on vegetation heights. The restoration project data are a mix of primary–i.e. observed and secondary–i.e. estimated costs and benefits (S3 Table). The coastal setting and exposure data for each project location are derived from other sources (see S1 Methods). Cost reporting by projects is highly variable (see Methods). All costs are reported on a per-m^2^ basis, and use total project costs for the cost comparison analyses. Ideally, in future, cost reporting in projects should be consistent and report both unit and total restoration costs. More such comparisons with hard alternatives, along with detailed and consistent data on the extents, costs and coastal protection benefits of existing restoration projects, are needed to inform the design and implementation of future nature-based defences.

We are interested in general conclusions about the parameters that influence wave reduction across multiple habitats and physical conditions. Therefore, the study uses average values of vegetation height and water depth for the parameter analyses. It is worth emphasising that the measurements of waves in the analysed studies are all under ‘normal’ conditions of low waves. Mean wave height values are used for the meta-analyses. Variations in wave height measurements at each site are accounted for within the analyses (see S1 Methods). However, when analysing extreme value measurements, it will be necessary to include analyses of variances to assess the effect on wave reduction. Also, site-specific variations in all these parameters will need to be considered when designing a nature-based defence project. For instance, the slope of a coral reef can influence variations in wave reduction over that reef [38] and hence, its effectiveness as a nature-based defence. Wave height is the response variable for the meta-analyses, following a number of the reviewed studies that report reductions in terms of wave heights. Field measurements and analyses of wave energy, rather than wave height, may provide a better picture of the processes that drive wave reduction at each site [21].

Field evidence of the protection offered by habitats is generally difficult to obtain. However, clear differentiation of measured parameters–i.e. physical reduction of wave heights or storm surges, versus economic savings in damage costs during extreme events–is essential to understand the extents to which, and conditions under which, different habitats offer protection. For instance, the review of nature-based defence projects suggests that mangroves are effective protection measures against flooding from storms (Table 1, S3 Table). The meta-analyses of wave heights however show that wave height measurements in mangroves have so far been limited to lower waves than in salt-marshes (Table 1, S1 Table).

Future studies of effectiveness and cost-effectiveness would also be strengthened by paired measurements of wave height reduction with and without habitat [39] accompanied by information on habitat parameters such as height, density and roughness [40,41,42]. A small but growing number of field observations, laboratory experiments and numerical models suggest that reefs and wetlands can act as buffers against extreme waves and water levels [8,43,44,45,46], though the observed data for extreme events is scant. It will also be critical to get similar field measurements of wave and water level reductions by habitats during extreme events [47]. When evaluating restoration projects for coastal protection, it would be useful to follow monitoring and evaluation procedures set out within established coastal engineering frameworks. These could usefully include demonstrations of projects implemented in different physical settings [20], theoretical design frameworks [48,49], or even, evaluations of nature-based defences within national accounts [37]. Such evaluation typically involves a before-after comparison of the coastal hazard at the site. However, a restoration project can typically have multiple objectives, the evaluation of which will require monitoring of outcomes at multiple impact and reference sites.

## Conclusions

This paper is, as far as the authors are aware, the first attempt at synthesising evidence from field measurements and restoration projects to provide an overview of the wave reduction by natural defences, in combination with site-specific comparisons of the costs of nature-based defences versus alternative structures. The paper also synthesises information on the benefits of restoration projects for coastal protection. These analyses and syntheses demonstrate the following: a) coastal habitats–particularly coral reefs and salt-marshes–have significant potential for reducing wave heights and providing protection at the shoreline; b) restoration projects for which data are available–i.e., mangrove and marsh projects–can be cost-effective relative to submerged breakwaters in attenuating low waves and become more cost-effective at higher water depths; c) a number of nature-based defence projects, especially in mangroves and marshes, have been observed to offer protection during storms. Variations in wave reduction and cost effectiveness are dependent on multiple parameters including water depth and vegetation / reef height.

Examples of nature-based defence projects are growing rapidly in number, but much better reporting of effectiveness and cost effectiveness is necessary, for better understanding of their viability. Data from post-project monitoring of the success or failure of restoration projects are not easily available. As with any hard engineering structure, information on nature-based defence project failures–i.e. the reasons why a particular project did not work can also be very valuable when developing guidelines and methodologies for project design. This would include, for instance, before and after observations of whether a restoration project designed for coastal protection has achieved its stated objectives. Ideally, project costs, site conditions and wave reduction extents should be measured at the same location. This will allow better understanding of variations in project costs with variations in water levels, wave conditions and habitat characteristics. This is particularly important for a future where variations in rates of sea-level rise and other environmental factors can result in a spatial variability in wave heights [50,51]. Also, better estimates of maintenance costs and the additional services and benefits (including coastal access, fish production, carbon sequestration) or lack thereof, for both artificial and nature-based defences are required when evaluating the overall costs and benefits of a restoration project that includes coastal protection as an objective. Finally, inclusion of dune and also beach habitats [52] would vastly improve the richness of existing nature-based defence databases.

## Methods

### Overview

The analyses of wave reduction measurements and restoration projects were conducted using two separate datasets with some overlap in habitat types. The wave reduction meta-analyses were performed for observations of wave heights in coastal habitats that provided information on wave heights with (before) and without (after) the habitat. In the meta-analyses, seagrass and kelp beds were treated together due to similarities in location and the mechanism by which they reduce wave heights (see Fig 1). The analyses of costs and benefits of nature-based defence projects were done for fifty-two restoration projects in coral reefs, oyster reefs, salt-marshes and mangroves, that were specifically targeted at coastal protection. Only studies that provided some quantitative information (observed or estimated) on project extents, costs and/or benefits were included in the analyses. The literature search did not find any projects within seagrass or kelp beds that met these criteria. Similarly, no wave reduction field measurements within oyster reefs were found. The cost-comparisons to alternative breakwaters were limited to habitats for which field measurement and project sites could be paired, which were only in mangroves and salt-marshes (see Nature-based defence project costs benefits and cost-effectiveness in this section).

### Natural defences and wave reduction

Broadly, wave reduction in habitats occurs by two mechanisms–(i) wave-breaking due to changes in water depth (i.e. in reefs) and; (ii) damping of wave energy and, hence, wave height through friction (i.e. in wetland habitats like mangroves, marshes or seagrass beds). This reduction in wave height depends on habitat and site-specific ecological and geophysical parameters that influence the dynamics of incoming waves (Fig 1). For instance, wave reduction in coral reefs is mainly influenced by: (i) the relative wave height, i.e. the ratio H/h where h is the depth of the reef and H the wave height; and (ii) the relative width, i.e. the ratio B/L, where B is the width of the reef and L the length of the incoming wave [53,54]. In vegetated habitats, the height, geometry and shoot/stem density of the habitat, have all been shown to affect wave reduction in flume studies and models [55,56,57]. A key parameter in intertidal vegetated habitats such as mangroves and marshes is the relative height of the vegetation i.e. the ratio h_v_/h, where h_v_ is the height of the vegetation canopy and h the water depth. In addition, these habitats are known to trap sediments [57,58], raising the near-shore bathymetry and thereby increasing their capacity to reduce waves. Wave heights within deeper vegetated habitats such as seagrass beds are also affected by changes in bathymetry [53].

Meta-analyses of the effect of habitat on wave reduction were done for sixty-nine studies in coral reefs, salt-marshes, mangroves, seagrass beds and kelp beds (Fig 2A), that provide measurements of wave height with /without coastal habitats. A literature search was performed in English for studies that describe measurements of wave reduction in coastal habitats, using Google Web, Google Scholar, Web of Science and other databases. Only studies that provide information on observed wave heights before (or, in front of) and after (or, behind) the habitat were included in the meta-analyses. The meta-analyses provide an aggregate assessment across multiple studies, of the effect that each habitat has on wave reduction. The two sub-tidal vegetated habitats, seagrass and kelp beds were treated together due to their similarities in wave reduction mechanisms (see Fig 1). For each habitat the effect of habitat presence on wave height reduction was measured using a random effects statistical model [59]; see S1 Methods). The response variable–namely, the reduction in wave height was expressed as a % of the incoming wave height (see S2 Table):

$$R=1-\frac{H_{st}}{H_{si}}$$ [1]

where H_i_ is the significant wave height (m) before the habitat (“incident”) and H_st_ is the significant wave height (m) after the habitat (“transmitted”). The rate of wave reduction per metre width of habitat was calculated as:

$$r=\frac{H_{si}-H_{st}}{B}=\frac{R*H_{si}}{B}$$ [2]

where B is the cross-shore width of the habitat (m) (i.e. length of the habitat transect). The average effect size was measured in terms of the log response ratio of wave height reduction due to the habitat. The averages were considered to be statistically significant if their 95% confidence intervals do not overlap zero (S5 Fig).

For studies that directly report incoming and transmitted wave heights (as opposed to studies that only report percentage reductions) we also showed the variation of absolute reduction extents versus incoming wave heights (see S2 Fig). However, for the analyses of design parameters, percentage reductions in wave heights were used to avoid compounding influences from other parameters. For this, average values of habitat widths, water depths and vegetation heights were extracted from the data. The average values of wavelength were obtained at each location from a global dataset of wave characteristics [60]. These were used to assess the response of wave height reduction to specific non-dimensional parameters: i) relative wave height H_i_/h, where h is the average water depth across the habitat transect; ii) relative width B/L, where L is the average annual deep-water wavelength at the habitat location and; iii) relative vegetation height h_v_/h in intertidal habitats where h_v_ is the average vegetation height across the transect. The first two parameters—H_i_/h and B/L are dependent on the incoming wave height. Therefore, studies that only report wave reduction ratios–i.e. do not report incident wave heights, were excluded for these parameters. The influence of bathymetry on wave height reduction was not accounted for, except where the study reported measurements from adjacent transects with and without the habitat. The extent to which bathymetry influences wave height reduction varies between habitat types and, in most cases, bathymetry is either a direct function of habitat presence (in reefs) or has a relatively minor influence on wave height reduction (in mangroves and salt-marshes).

### Nature-based defence projects: costs, benefits and cost-effectiveness

The analyses of costs and benefits of restoration projects were done for fifty-two projects in coral reefs, salt-marshes, mangroves and oyster reefs. An initial systematic search was conducted for peer-reviewed literature and grey literature (e.g. reports, assessments, surveys, etc.) on the coastal protection and risk reduction costs and benefits of projects involving restoration and management of coastal habitats. The search was conducted in English on the Google Web and Google Scholar databases. We only searched for projects that were targeted at coastal protection and reported sufficient information on costs and habitat characteristics for further analyses (see S1 Methods, S3 Table). Studies that did not deal with coastal protection as a stated objective were excluded. Studies that did not report data on either costs or benefits were also excluded. From the fifty-two projects, a subset was identified that also reported observed and estimated coastal protection benefits of various types. Cost reporting in the project dataset is highly variable: of the 52 projects, fourteen do not report any costs, seventeen report total restoration costs, nine report costs on a per-m^2^ basis, nine on a per-hectare basis, two as per-metre coastline length and one as per-kilometre coastline length (S3 Table). All costs were summarised on a per m^2^ basis. All reported monetary values were standardised to 2014 US$ equivalents by inflating these from the project year to 2014 using appropriate Consumer Price Index (CPI) inflator indices and converting the inflated costs to 2014 US $ [61,62].

To provide a direct comparison of restoration projects with engineering alternatives, the costs of restoration projects were compared to the costs of structures that would achieve the same wave reduction. We were unable to find projects that reported specific comparisons to coastal structures or other measures of effectiveness (e.g., reduction of waves, erosion rates or flood volumes). Ideally, in future, more demonstration and reference sites would be available at multiple scales, to be able to compare the costs and effectiveness of nature-based defences versus artificial structures [27]. Submerged breakwaters were chosen for the cost comparisons since these structures perform similarly with regard to reduction of wave heights at the coastline. It is recognised that restoration costs do not vary linearly with habitat size. Therefore, information on restoration costs was combined with data from nearby measurements of wave heights to estimate the wave reduction benefits of each restoration project. The cost of breakwater needed to achieve the same wave reduction benefits in that location was then calculated. All costs are presented on a per metre coastline length basis.

For pairing the project sites with field measurements, thirteen unique pairs were identified that occur a) at close proximity; b) in similar coastal setting, e.g. habitat. Sites were paired if they were within 50 km of each other. In some cases, a project site could be paired with multiple field measurements (see S4 Table). All criteria for pairing sites were visually inspected and based on expert judgement (see S4 Table). For each pair of project and field measurement, the per metre project cost (*C*_*proj_per_m*_) and width (*B*_*proj*_) were transferred to the field measurement location and a nature-based defence (NbD) was defined with a unique combination of width (*B*_*NbD*_), cost (*C*_*NbD*_), rate of wave height reduction (*r*_*NbD*_), incoming wave height (*H*_*NbD*_) and water depth (*h*_*NbD*_):

$$C_{NbD}=C_{proj\_per\_m}\;(from\;project)$$ [3]

$$B_{NbD}=B_{proj}\;(from\;project)$$ [4]

$$r_{NbD}=r\;(from\;field\;measurement)$$ [5]

$$H_{NbD}=H_{si}\;(from\;field\;measurement)$$ [6]

$$h_{NbD}=h\;(from\;field\;measurement)$$ [7]

Using the rate of reduction and project width the total wave reduction by each NbD was estimated and expressed as a transmission coefficient, *K*_*t*–*NbD*_. Using Eqs [1] and [2],

$$K_{t-NbD}={\left(\frac{H_{st}}{H_{si}}\right)}_{NbD}=1-\frac{r_{NbD}*B_{NbD}}{H_{i}}$$ [8]

For each replacement breakwater the minimum crest-width, W and freeboard, F (i.e. crest height relative to water surface) required to achieve the same transmission coefficient as the NbD, K_t-nbD_ were calculated. The breakwater was assumed to have a trapezoidal section with a representative slope, s of 1:1.5 (S6 Fig). Breakwater dimensions were computed per metre coastline using standard coastal engineering formulae [53]; see S1 Methods: Eqs [SI 11]–[SI 13]). Using the estimated values of freeboard F, water depth, h, crest width W and slope, s the volume of the breakwater was calculated as:

$$V_{struc}=0.5*(h+F)*(W+W+(h+F)/s)$$ [9]

Next the unit construction cost–i.e. construction cost per metre length of coastline–of 1 cubic metre of breakwater was estimated as:

$$C_{struc\_unit}=C_{rep}/V_{rep}$$ [10]

where *V*_*rep*_ is the volume of the representative breakwater per metre coastline and *C*_*rep*_ is the total construction cost per metre coastline of this breakwater ([29]; see S1 Methods: Eqs [SI 15]–[SI 16]). The breakwaters were assumed to be constructing using rock or rubble-mound, as this is the most commonly employed material world-wide. Breakwater construction cost was assumed to be proportionate to the size of the structure [63,64], and using Eqs [9] and [10] the cost per metre coastline length was estimated for each replacement breakwater,

$$C_{struc}=V_{struc}*C_{struc\_unit}$$ [11]

where, V_struc_ is the volume per metre coastline of the breakwater and C_struc_unit_ the unit construction cost of one cubic metre of breakwater, per metre coastline. A replacement cost ratio for each NbD based on the cost of the replacement breakwater, C_struc_ and the cost of the NbD, C_NbD_ was then calculated:

$$RCratio=\frac{C_{struc}}{C_{NbD}}$$ [12]

Construction costs for breakwaters in Vietnam were assumed to be ten times less than in Europe and the USA due to lower labour and material costs [28]. Since structure costs are critically dependent on water depth we also generated cost curves for breakwater construction at different water depths for a fixed wave height of 0.2 m–the average wave height across all NbD sites, and plotted these together with NbD costs (Fig 3). In estimating breakwater costs, a constant representative crest width, W of 2 m was assumed.

## Supporting Information

S1 Fig Absolute wave reduction versus wave heights. Absolute wave reduction extents are plotted against incident wave height for a) coral reefs (n = 27); b) mangroves (n = 11); c) salt-marshes (n = 14); d) seagrass/kelp beds (n = 5). This plot excludes measurements that do not report incoming wave heights.
(EPS)

S2 Fig Percentage wave reduction versus habitat width. Field measurements of % wave height reduction are plotted versus habitat width for a) coral reefs (n = 34); b) mangroves (n = 14); c) salt-marshes (n = 15); d) seagrass/kelp beds (n = 6). Significant relationship found only for coral reefs.
(EPS)

S3 Fig **Percentage wave height reduction versus a) relative wave height and b) relative width in coral reefs.** Field measurements of % wave height reduction are plotted versus non-dimensional engineering parameters: (a) Hi/h in reefs (left, n = 27), red line indicates depth-limiting ratio for wave height, Hi/h = 0.78; (b) B/L in coral reefs (right, n = 34). Plot (b) shown for the blue region in inset. Red circle indicates outlier points excluded in regression analyses (see S1 Methods).
(TIF)

S4 Fig Percentage wave height reduction versus relative height of salt-marshes. Field measurements of % wave height reduction versus non-dimensional parameter, h_v_/h in salt-marshes (n = 8). Red line indicates relative vegetation height h_v_/h = 1, below which the vegetation is fully submerged. One point (circled in red) with very low relative height and very high wave attenuation was excluded as an outlier for the regression analysis (see S1 Methods). We do not perform regression analyses for mangroves and seagrass/kelp beds due to insufficient information on engineering parameters for these habitats.
(TIF)

S5 Fig Log response ratio of wave reduction effect size by habitat type. Average effect size as log response ratio of the wave reduction, R due to each habitat type for coral reefs, salt-marshes, mangroves and seagrass/kelp beds. Dots represent average values and error bars represent 95% Confidence Intervals). The averages are considered significant (p<0.05) when the error bars do not overlap zero (see S1 Methods). The number of independent studies analysed is indicated in brackets.
(EPS)

S6 Fig Cross-section of a rubble-mound breakwater. Simplified submerged breakwater cross-section for replacement cost estimates, showing parameters that affect wave transmission. Fig is adapted from van der Meer et al. (2005) and US Army Corps of Engineers (2015b).
(TIF)

S1 Methods Supplementary Methods. See file “S1 Methods.”
(DOCX)

S1 Table Wave reduction percentages, habitat and site properties for different habitat types (see Fig 1 for parameter definitions). n = total number of field measurements for each habitat. Values in brackets indicate 95% confidence intervals.
(TXT)

S2 Table Wave height, habitat conditions and site condition measurements. Metadata included within file.
(TXT)

S3 Table Project data on habitat type, conditions, project extents, costs and benefits. Metadata included within file.
(TXT)

S4 Table Project–Field Measurement Pairs for Replacement Cost Ratio Analyses. Metadata included within file.
(TXT)
