# Supplementary figures and images for: The Effectiveness, Costs and Coastal Protection Benefits of Natural and Nature-Based Defences
Source: PLoS One. 2016 May 2;11(5):e0154735. doi: 10.1371/journal.pone.0154735 (PMC4852949; doi:10.1371/journal.pone.0154735)

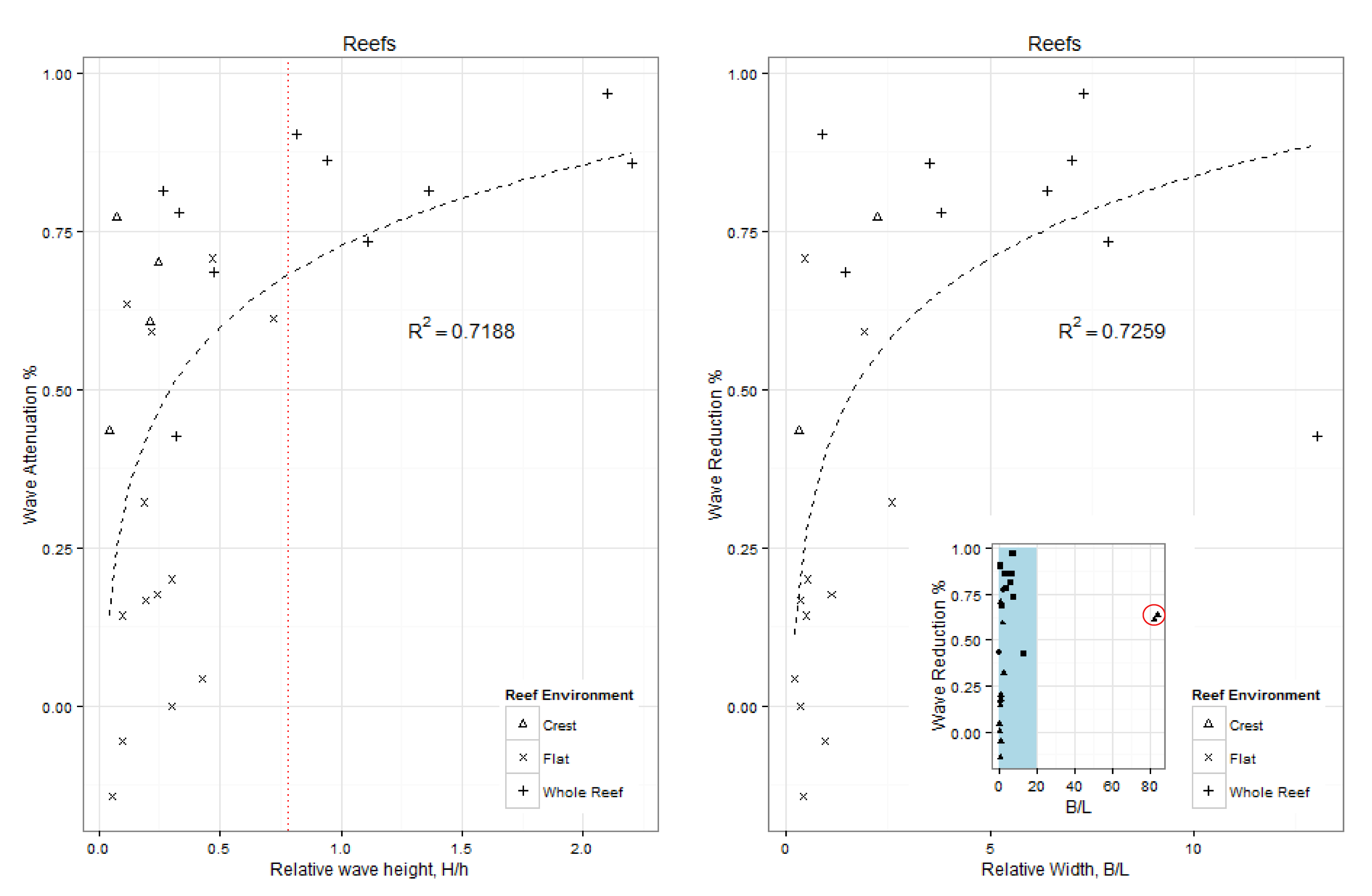

Supplement: S3 Fig — Percentage wave height reduction versus a) relative wave height and b) relative width in coral reefs. Field measurements of % wave height reduction are plotted versus non-dimensional engineering parameters: (a) Hi/h in reefs (left, n = 27), red line indicates depth-limiting ratio for wave height, Hi/h = 0.78; (b) B/L in coral reefs (right, n = 34). Plot (b) shown for the blue region in inset. Red circle indicates outlier points excluded in regression analyses (see S1 Methods). (TIF) [file pone.0154735.s003.tif]

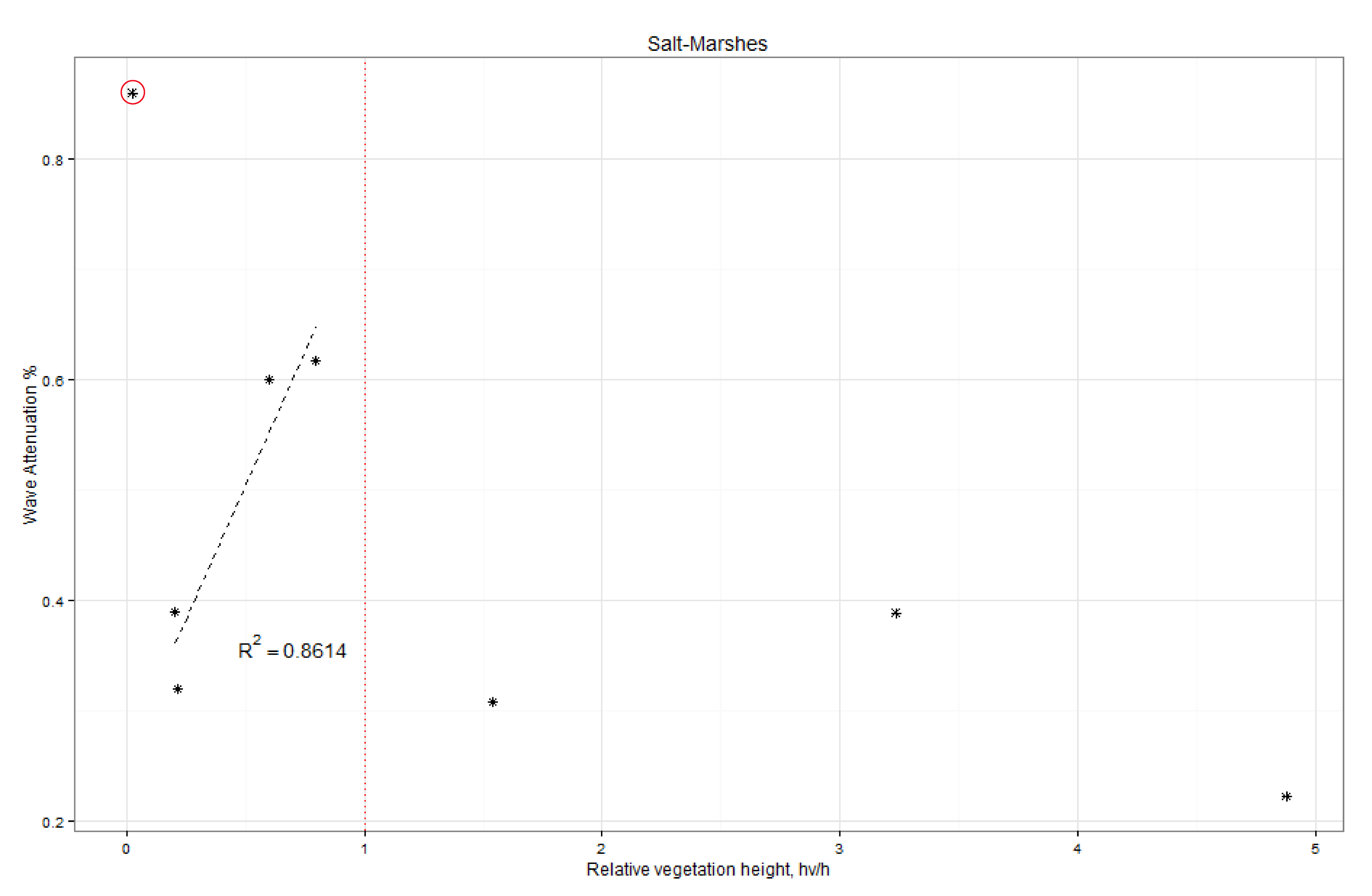

Supplement: S4 Fig — Field measurements of % wave height reduction versus non-dimensional parameter, hv/h in salt-marshes (n = 8). Red line indicates relative vegetation height hv/h = 1, below which the vegetation is fully submerged. One point (circled in red) with very low relative height and very high wave attenuation was excluded as an outlier for the regression analysis (see S1 Methods). We do not perform regression analyses for mangroves and seagrass/kelp beds due to insufficient information on engineering parameters for these habitats. (TIF) [file pone.0154735.s004.tif]

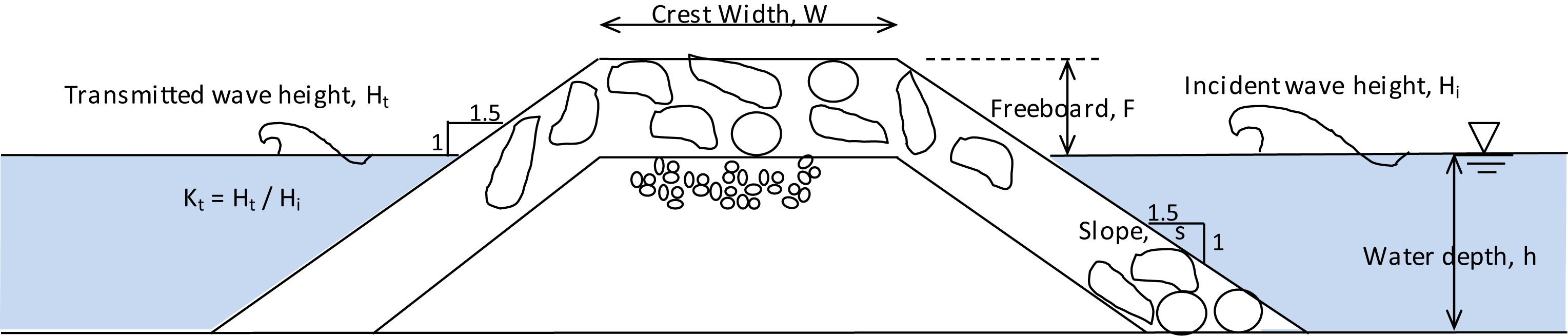

Supplement: S6 Fig — Simplified submerged breakwater cross-section for replacement cost estimates, showing parameters that affect wave transmission. Fig is adapted from van der Meer et al. (2005) and US Army Corps of Engineers (2015b). (TIF) [file pone.0154735.s006.tif]
