# Supplementary material for: The Effectiveness, Costs and Coastal Protection Benefits of Natural and Nature-Based Defences
Source: PLoS One. 2016 May 2;11(5):e0154735. doi: 10.1371/journal.pone.0154735 (PMC4852949; doi:10.1371/journal.pone.0154735)
Supplement: S1 Methods — See file “S1 Methods.” (DOCX) [file pone.0154735.s007.docx]

### SI Methods

#### Wave height reduction in coastal habitats: syntheses and analyses of global field evidence

Literature search: The wave height reduction meta-analyses synthesise data from field measurements of wave heights in coastal habitats. A literature search was performed in English, using Google Web and Google Scholar databases for studies that describe measurements of wave reduction in coastal habitats. Search terms included <habitat type> + <wave reduction type> where <habitat type> is either “mangrove”, “marsh”, “wetland”, “coral reef”, oyster reef”, “seagrass” or “kelp” and <wave reduction type> is “wave height”/”wave energy” + “reduction” /”attenuation” /”spending” /”mitigation”. Two wave reduction measurements in fresh water marshes were included with the salt-marsh studies. The same search was performed on the Web of Science database and other databases for studies that may have been missed. These included Building with Nature in the Netherlands, Managed Realignment in the UK, Living Shorelines and Engineering with Nature, case-studies in the USA [1-4] and also previous syntheses on reefs [5] and salt-marsh wetlands [6, 7]. The bibliography and reference lists of selected literature were also searched for relevant studies.

Meta-analyses procedure: We performed meta-analyses of the influence of habitat presence on wave height reduction. The studies were divided into four habitat groups – coral reefs, mangroves, salt-marshes and seagrass/kelp beds and each group analysed separately. From the meta-analyses the average wave height reduction values for each habitat group were reported along with their 95% confidence intervals. A random effects model was used for each group, since these studies use different techniques, instruments and protocols for measuring wave height transformation. In a random effects model, the true effects in the studies are assumed to have been sampled from a distribution of true effects and the summary effect is an estimate of the mean of all relevant true effects, with the null hypothesis being that the mean of these effects is one, and the log response ratio is zero. The random effects model uses a weighted mean, where the weight assigned to each study is the inverse of the total variance which is calculated as the sum of the in-study variance and an estimate of the between-studies variance [8]. The overall wave height reduction for each habitat group was obtained as the inverse logarithm of the effect size of its random effects model and expressed as a percentage. The effect size was calculated as the log-response ratio (ln R) and its corresponding weighting factor, calculated as the reciprocal of the variance in the random effects model. The overall effect size (ln R) was then obtained by summing the products (ln R X weighting factor) and dividing it by the sum of the weights. For each response variable the overall effect size was considered statistically significant if its 95% confidence interval did not include zero (see S5 Fig).

To estimate the mean effect size for each habitat using this model the following values were first extracted from each study:

1. Mean incident wave height (wave height before/without the habitat), H_si_ and associated error
2. Mean transmitted wave height (wave height after/with the habitat), H_st_ and associated error
3. Number of identifiably independent measurements for each study
4. Habitat width (length of measurement transect), B

The response variable (R) is the wave height reduction relative to the incident wave height expressed as

Wave height reduction, $R=1- \frac{H_{t}}{H_{i}}$ [SI 1]

where H_t_ is the transmitted wave height (m), H_i_ is the incident wave height (m). A rate of wave height reduction, r over the habitat per metre coastline length was also estimated as:

$r= \frac{H_{i}-H_{t}}{B}$ [SI 2]

where, B is the cross-shore width of the habitat (m) (i.e. length of the measurement transect). The ratio H_t_/H_i_ is often referred to in coastal engineering design as the transmission coefficient, K_t_, such that:

$K_{t}=\frac{H_{st}}{H_{si}}=1-\frac{r*B}{H_{si}}$ [SI 3]

Data extraction and variable treatment: The mean values of wave heights were either directly extracted from the study or calculated from a value range as reported in each study. Similarly, incident and transmitted wave height error values for the random effects model were extracted either directly or as the standard deviation of a range of wave height values. Where error values were not available these were assumed as 10% of the measured value [9, 10]. The number of measurements was taken as the number of distinct sampling groups reported by the study. For all analyses we used the significant wave height, H_s_, the standard wave parameter for engineering measurements and design. For studies that report wave energies these were converted to wave heights using the formula

$E= \frac{1}{8}\rho gH_{s}^{2}$ [SI 4]

where E is the wave energy (J/m^2^), rho, the density of sea-water (kg/m^3^), g, gravitational acceleration (m/s^2^) and H_s_, the significant wave height (m).

For coral reefs, the incident and transmitted wave heights are the wave heights before and after the reef, respectively. Where studies reported measurements for the whole reef along with measurements for reef crest or flat we considered only the whole reef measurements to avoid duplication. For the seagrass/kelp habitats and the intertidal habitats (i.e. marshes and mangroves) as well, the incident and transmitted wave heights were the wave heights before and after the habitat, except where information from adjacent transects with and without the habitat was available. In these cases, the wave height on a transect without the habitat was the incident wave height and the wave height on an adjacent transect with the habitat was the transmitted wave height. Bathymetry, i.e. water depth, can critically influence wave height reduction extents, and we accounted for variations in bathymetry wherever data from adjacent transects were available. In most cases, bathymetry is either a direct function of habitat presence, such as in coral reefs; or it has a negligible influence on wave height reduction such as in mangroves and salt-marshes where bottom slopes are typically mild [11, 12]. The influence of bathymetry may be more pronounced in seagrass beds: of the six seagrass/kelp studies, three studies consider bathymetry (see SI Table 2: “Depth_Control”).

All analyses were done in R and RStudio. Mapping was done in ArcGIS and data grabs from graphs were done using a graph extraction code in Matlab [13].

Trend analyses of biophysical parameters: We analysed the response of wave height reduction, R to biophysical parameters and for specific habitats using a generalised linear model. We assessed the following parameters and habitats :

1. Relative wave height, H_i_/h (i.e. ratio of incident wave height over water depth)
2. Relative width, B/L (i.e. ratio of habitat width over deep-water wavelength)
3. Relative vegetation height, h_v_/h for intertidal habitats (salt-marshes and mangroves) (i.e. ratio of vegetation height over water depth)

From each study average values of habitat widths and water depths and vegetation heights were extracted. Since incident wave heights will influence wave transformation within a habitat, studies that do not report incident wave heights, but only report wave height/energy reduction ratios, were excluded from all regression analyses. Two exceptionally wide coral reef data points (S2 Fig) and one exceptionally low salt-marsh data point (S3 Fig), were disregarded as outliers for the analyses of the parameters B/L and h_v_/h, respectively. The deep-water wavelength for the parameter B/L was estimated using wave period data from a global wave climate re-analysis database for each study site [14].

#### Assessment of costs and coastal protection benefits of habitat restoration projects

Literature search: An initial systematic search was conducted for peer-reviewed literature and grey literature (e.g. reports, assessments, surveys, etc.) on the coastal protection and risk reduction costs and benefits of projects involving restoration and management of coastal habitats. The search was conducted in English on the Google Web and Google Scholar databases. The search terms used were a combination of <habitat type> + <coastal protection costs and benefits>. The <habitat type> search included “coral reefs”, “oyster reefs”, “mangroves”, “salt-marshes”, “wetlands”, “seagrass” and “kelp”. The <coastal protection costs and benefits> search included “coastal protection”, ”flood reduction”, ”risk reduction”, ”erosion control” + “costs”, ”benefits”. Studies that did not deal with coastal protection as a stated objective were excluded. Studies that did not report data on either costs or benefits were also excluded. 52 projects were found that provide some measure of coastal protection costs and/or benefits. 39 of the 52 projects report quantitative measurements of project costs.

Review and analyses of costs, benefits, benefit-cost ratios and cost effectiveness ratios: Information on observed benefits from each study was classified into 3 types – reductions in coastal erosion rates, flood damages or engineering costs for coastal defence structures. The number of projects that report each type of benefit as counted, as was the number of projects that state coastal protection as their primary objective. Each habitat type was treated separately in calculating mean and median values, 95% confidence intervals and standard error values for costs and benefit-cost ratios. For the cost effectiveness analyses we used information on total project costs to derive a representative ‘cost per metre length of coastline’ for each project, to enable comparison with coastal structures. The cost per metre length of coastline for each project was calculated as:

$C_{proj\_per\_m}=\frac{C_{proj}}{L_{proj}}=\frac{C_{proj}}{{(A}_{proj}/B_{proj})}$ [SI 5]

where, C_proj_ is the total restoration cost, L_proj_ is the along-shore length of the project, A_proj_ the total project area and B_proj_ the cross-shore width of the habitat.

Where data on project areas and widths were unavailable these were obtained from Google Earth based on the project location. All reported costs are standardised to 2014 US$ equivalents using the appropriate exchange rates and Consumer Price Index (CPI) inflator indices [15, 16].

#### Replacement cost ratio analyses for nature-based defence projects

Step 1: Project extent and cost per m coastline length: From all the project and field measurements we identified thirteen unique data that occur a) at close proximity; b) in similar coastal setting, e.g. habitat. Sites were paired if they were within 50 km of each other. In some cases a project site could be paired with multiple field measurements (see S4 Table). All criteria for pairing sites were visually inspected and based on expert judgement.

Due to the lack of measurements of costs and physical parameters at the same site it was assumed that the cost and habitat characteristics from the project site are applicable at the field measurement location. We used project costs as reported and estimated the wave reduction for the project extent based on the measured rate of reduction nearby. Each project’s restoration cost was expressed in terms of a ‘per metre length of coastline’ to enable comparison with unit structure costs. Thus, assuming that the project is located at the field site, the project cost ($C_{proj\_per\_m}$ ) and width ($B_{proj})$ were transferred to the field measurement location and a unique nature-based defence (NbD) was defined in terms of width ($B_{NbD})$, cost ($C_{NbD}$), rate of wave height reduction ($r_{NbD}$), incoming wave height ($H_{NbD}$) and water depth ($h_{NbD}$):

$C_{NbD}=C_{proj\_per\_m}$ [SI 6]

$B_{NbD}=B_{proj}$ [SI 7]

$r_{NbD}=r$ [SI 8]

$H_{i-NbD}=H_{i}$ [SI 9]

$h_{NbD}=h$ [SI 10]

Step 2: Total wave reduction and transmission coefficient by nature-based defence: The total wave height reduction by each NbD was estimated and expressed in terms of a transmission coefficient using equation [SI 3],

$K_{t-NbD}=1-\frac{r_{NbD}*B_{NbD}}{H_{i}}$ [SI 11]

Step 3: Dimensions and volume of replacement breakwater for K_t-NbD_: For each NbD, the minimum relative freeboard and crest-width of a submerged breakwater were estimated that will achieve the same transmission coefficient as the NbD under the same water depth and wave height conditions. These dimensions were computed per metre coastline using standard coastal engineering formulae for K_t_ in submerged breakwaters [17]:

$K_{t-NbD}=K_{t-struc}= -0.40\frac{F}{H_{NbD}}+0.64\left( \frac{W}{H_{NbD}} \right)^{-0.31}(1-e^{-0.50\varepsilon_{op}})$ [SI 12]

for $\frac{W}{H_{NbD}}<8$, with imposed lower and upper limit values of 0.05 and 0.75, and

$K_{t-NbD}=K_{t-struc}= -0.35\frac{F}{H_{NbD}}+0.51\left( \frac{W}{H_{NbD}} \right)^{-0.65}(1-e^{-0.41\varepsilon_{op}})$ [SI 13]

for $\frac{W}{H_{NbD}}>12$ with imposed lower and upper limit values of 0.05 and $0.93-006(\frac{W}{H_{NbD}}$), with values for $\frac{W}{H_{NbD}}$ between 8 and 12 obtained by linear interpolation.

where, F is the freeboard (i.e. crest height relative to water surface), W is the crest width for the breakwater, H_NbD_ is the design wave height and $\varepsilon_{op}$ is the surf similarity parameter calculated based on the peak deep water wave period at each site and an assumed bed slope of 1:100. F and W were calculated for the desired value of K_t-struc_. From the water depth, h and freeboard F the volume (m^3^) per metre coastline of a trapezoidal structure is calculated assuming a representative structure slope of 1:1.5 (see S6 Fig):

$V_{struc}=0.5*\left( h+F \right)*(W+W+\left( h+F \right)/s)$ [SI 14]

Step 4: Breakwater cost and replacement cost ratio: First unit construction costs were estimated using the volume and costs of a representative armoured rubble-mound breakwater from coastal engineering literature. Based on the total representative volume ($V_{rep}$) and per metre costs ($C_{rep}$) of this cross-section, we calculated a unit construction cost per metre coastline per cubic metre of submerged breakwater ($C_{struc\_unit}$):

$C_{struc\_unit}=C_{rep}/V_{rep}$ [SI 15]

A $V_{rep}$ of 36 m^3^ and a $C_{rep}$ of 2700 US $ per metre were obtained, giving a $C_{struc\_unit}$ of around 761 US $ per m^3^ per metre are chosen from the literature (breakwater cross-section and cost data from ]18]: Fig VIII-21 and Table VIII-20). Assuming that construction costs vary proportionate to breakwater volume, the total cost per metre coastline length for each replacement breakwater ($C_{struc}$) was obtained as:

$C_{struc}=V_{struc}*C_{struc\_unit}$ [SI 16]

where $V_{struc}$ is the volume per metre coastline of the replacement breakwater. Unit construction costs for breakwaters in Vietnam were assumed to be ten times less than in Europe and the USA [19]. A replacement cost ratio for each NbD – structure pair (Table 1) were also obtained:

$RCratio=\frac{C_{struc}}{C_{NbD}}$ [SI 17]

Since structure costs are critically dependent on water depth, breakwater cost curves versus water depth were generated for an incoming wave height of 0.2 m – the average wave height across all the NbDs, and plotted together with the costs of the NbDs at different depths (see Results; Fig 3). For the cost curve calculations a constant breakwater crest-width, W of two metres was assumed.

There is a concurrent effort to make the databases of field measurements and nature-based defence projects more comprehensive and easily accessible. This includes an on-going exercise to collect, map and integrate qualitative and quantitative information on the physical effectiveness and cost effectiveness of nature-based defence projects globally. This is available as an interactive, online database with the aim of providing readily available information on different habitat and project types to coastal managers around the world (<http://maps.coastalresilience.org/global> - “Natural Defence Projects’ tab; [20]). The database draws from multiple sources similar to the analyses discussed here (see SI Methods) and is aimed to facilitate the future addition of relevant information on the effectiveness, costs and benefits of any project that restores or manages a coastal habitat for coastal protection and/or risk reduction.

#### References

1. de Vriend H & Van Koningsveld M (2012) Building with Nature. *Thinking, acting and interacting differently. Dordrecht, the Netherlands: Ecoshape, Building with Nature*.

2. ABPmer (2014) Online Marine Registry (OMReg): Adapting shorelines.

3. COPRI (2014) Coasts, Oceans, Ports and Rivers Institute: Living Shorelines Database.

4. US Army Corps of Engineers (2015) Engineering with Nature. (US Army Corps of Engineers Environmental Laboratory).

5. Ferrario F*, et al.* (2014) The effectiveness of coral reefs for coastal hazard risk reduction and adaptation. *Nat Commun* 5.

6. Shepard CC, Crain CM, & Beck MW (2011) The protective role of coastal marshes: a systematic review and meta-analysis. *PLoS ONE* 6(11):e27374.

7. Gedan KB, Kirwan ML, Wolanski E, Barbier EB, & Silliman BR (2011) The present and future role of coastal wetland vegetation in protecting shorelines: answering recent challenges to the paradigm. *Climatic Change* 106(1):7-29.

8. Borenstein M, Hedges L, Higgins J, & Rothstein H (2005) Comprehensive meta-analysis version 2. *Englewood, NJ: Biostat* 104.

9. Janssen PA, Abdalla S, Hersbach H, & Bidlot J-R (2007) Error estimation of buoy, satellite, and model wave height data. *Journal of Atmospheric and Oceanic Technology* 24(9):1665-1677.

10. Dobson E, Monaldo F, Goldhirsh J, & Wilkerson J (1987) Validation of Geosat altimeter‐derived wind speeds and significant wave heights using buoy data. *Journal of Geophysical Research: Oceans (1978–2012)* 92(C10):10719-10731.

11. Burger B (2005) Wave attenuation in mangrove forests. (TU Delft, Delft University of Technology).

12. De Vos W (2004) Wave attenuation in mangrove wetlands. Red River Delta, Vietnam. (TU Delft, Delft University of Technology).

13. Doke J (2007) GRABIT - MATLAB Release. (The Mathworks, Inc.).

14. Reguero B, Menéndez M, Méndez F, Mínguez R, & Losada I (2012) A Global Ocean Wave (GOW) calibrated reanalysis from 1948 onwards. *Coastal Engineering* 65:38-55.

15. XE (2014) XE Live Exchange Rates.

16. FXTOP (2014) Inflation calculator and change of price between 2 dates.

17. van der Meer JW, Briganti R, Zanuttigh B, & Wang B (2005) Wave transmission and reflection at low-crested structures: Design formulae, oblique wave attack and spectral change. *Coastal Engineering* 52(10):915-929.

18. US Army Corps of Engineers (2015) North Atlantic Coast Comprehensive Study Report: Appendix T Coastal Storm Risk Management Measures. (US Army Corps of Engineers North Atlantic Division).

19. Hillen M*, et al.* (2010) Coastal defence cost estimates: Case study of the Netherlands, New Orleans and Vietnam. (TU Delft, Department Hydraulic Engineering).

20. SNAP Coastal Defenses Working Group (2014) Natural Defense Projects (SNAP Coastal Defenses Working Group).
